# Stress and neuroinflammation: a systematic review of the effects of stress on microglia and the implications for mental illness

**DOI:** 10.1007/s00213-016-4218-9

**Published:** 2016-02-05

**Authors:** Marilia A. Calcia, David R. Bonsall, Peter S. Bloomfield, Sudhakar Selvaraj, Tatiana Barichello, Oliver D. Howes

**Affiliations:** Department of Psychosis Studies, Institute of Psychiatry, Neurology and Neuroscience (IoPPN), King’s College London, Denmark Hill, London, SE5 8AZ UK; Psychiatric Imaging Group, Imperial College, MRC Clinical Sciences Centre, Hammersmith Hospital, London, W12 0NN UK; Department of Psychiatry and Behavioural Sciences, The University of Texas Health Science Centre at Houston, Houston, TX 77054 USA

**Keywords:** Stress, Inflammation, Microglia, Psychosis, Neuroinflammation

## Abstract

**Rationale:**

Psychosocial stressors are a well-documented risk factor for mental illness. Neuroinflammation, in particular elevated microglial activity, has been proposed to mediate this association. A number of preclinical studies have investigated the effect of stress on microglial activity. However, these have not been systematically reviewed before.

**Objectives:**

This study aims to systematically review the effects of stress on microglia, as indexed by the histological microglial marker ionised calcium binding adaptor molecule 1 (Iba-1), and consider the implications of these for the role of stress in the development of mental disorders.

**Methods:**

A systematic review was undertaken using pre-defined search criteria on PubMed and EMBASE. Inclusion and data extraction was agreed by two independent researchers after review of abstracts and full text.

**Results:**

Eighteen studies met the inclusion criteria. These used seven different psychosocial stressors, including chronic restraint, social isolation and repeated social defeat in gerbils, mice and/or rats. The hippocampus (11/18 studies) and prefrontal cortex (13/18 studies) were the most frequently studied areas. Within the hippocampus, increased Iba-1 levels of between 20 and 200 % were reported by all 11 studies; however, one study found this to be a duration-dependent effect. Of those examining the prefrontal cortex, ∼75 % found psychosocial stress resulted in elevated Iba-1 activity. Elevations were also consistently seen in the nucleus accumbens, and under some stress conditions in the amygdala and paraventricular nucleus.

**Conclusions:**

There is consistent evidence that a range of psychosocial stressors lead to elevated microglial activity in the hippocampus and good evidence that this is also the case in other brain regions. These effects were seen with early-life/prenatal stress, as well as stressors in adulthood. We consider these findings in terms of the two-hit hypothesis, which proposes that early-life stress primes microglia, leading to a potentiated response to subsequent stress. The implications for understanding the pathoaetiology of mental disorders and the development of new treatments are also considered.

## Introduction

Large-scale epidemiological studies have shown that stress, both early in childhood and later in life, predisposes to the development of mental health problems in adulthood (Scott et al. 2012; Varese et al. 2012; Benjet et al. 2010; Kessler et al. 2010). Several hypotheses, including alterations in the hypothalamic-pituitary stress system, abnormal immunological responses and lasting changes in cellular, molecular and epigenetic forms of plasticity, have been proposed to explain the neurobiological pathways that link childhood adversities to later development of adult mental illnesses.

The immune system responds to stressors and communicates with the central nervous system through a number of mechanisms, including cytokine signalling, vagal innervation and the lymphatic system (Louveau et al. 2015; Dantzer et al. 2008; Wan et al. 1994). It has been reported that stressful life experiences are associated with elevated pro-inflammatory cytokines in childhood and are also associated with the high risk of mental illness in adulthood (Khandaker et al. 2014; Danese et al. 2008). Such cytokine elevations may induce changes to cortical microglia, which in turn may be associated with structural and functional changes in the brain that predispose individuals to mental illness (see review by Réus et al. 2015). Supporting this, changes in microglial markers have been reported in a number of mental disorders such as depression (Torres-Platas et al. 2014), anxiety (Frick et al. 2013), schizophrenia (van Berckel et al. 2008) and autism spectrum disorders (Réus et al. 2015; Morgan et al. 2012). A recent PET imaging study demonstrated increased microglial activity in patients with schizophrenia and persons who are at ultra high risk of psychosis. Furthermore, greater microglial activity was positively correlated with greater symptom severity in the at-risk population (Bloomfield et al. 2015), suggesting a link between neuroinflammation and risk of psychosis.

Microglia are myeloid cells which provide the main form of adaptive immune response in the central nervous system (CNS). These cells modulate neuronal function not only during an inflammatory response but also during developmental synaptic pruning (Paolicelli et al. 2011) and plasticity in the healthy brain (Parkhurst et al. 2013; Tremblay et al. 2010) and are able to rapidly respond to even minor changes in the brain (Lawson et al. 1990). Microglia monitor the functional state of synapses, influence neuroplastic changes by remodelling extracellular spaces and eliminate synaptic elements by phagocytosis (Brown and Neher 2014; Kettenmann et al. 2013; Schafer and Stevens 2010).

In response to harmful stimuli, microglia undergo a number of changes (Walker et al. 2014). These include an increase in number due to proliferation (Kettenmann et al. 2011) and through recruitment of monocytes from the peripheral blood (Wohleb et al. 2013). Production of pro-inflammatory cytokines and the expression of several cell surface antigens are also features of inflammatory microglial response (Table 1). Of these, Iba-1 has been widely used to study microglia as its expression is specific and is expressed by both reactive and quiescent microglial cells (Frick et al. 2013).

**Table 1:** Common surface antigen markers of microglia and other centrally active immune cells

Marker	Function	Microglial significance
CD68	Involved in phagocytosis (Ramprasad et al. 1996)	Localised to monocytes and neutrophils (Saito et al. 1991)
CD11b (complement receptor 3)	Regulates leukocyte adhesion and migration to mediate inflammatory response (Meerschaert and Furie 1995)	Expressed in neutrophils, monocytes, natural killer cells, specific lymphocytes (Arnaout 1990)
Ionised calcium binding adapter molecule 1 (Iba-1)	Role in membrane ruffling and phagocytosis (Ohsawa et al. 2000)	Expressed centrally by microglia and infiltrating macrophage (Wohleb et al. 2013, Ito et al. 1998)
CD45	Modulates activation and proliferation of inflammatory cell types (Huntington and Tarlinton 2004)	CD45_low_—quiescent microglia
		CD45_high_—peripheral monocytes (Denker et al. 2007)
IL-1β, TNF-α, IL-6	Pro-inflammatory cytokines	Released by stimulated neutrophils and monocytes (Konsman et al. 2002)
MHC-II	Role in antigen presentation to T cells (St Pierre and Watts 1991)	Expressed centrally by stimulated microglia and macrophage (Aloisi 2001; Xu and Ling 1994)
CCL2/MCP-1	Triggers microglial proliferation and recruits other pro-inflammatory cells (Hinojosa et al. 2011)	Produced by stimulated microglia and expressed in high levels by infiltrating macrophage (Selenica et al. 2013)
CX3CR1 (fractalkine receptor)	Role in leukocyte migration and adhesion (Imai et al. 1997)	CCR2_low_/CX3CR1_high_—microglia CCR2_high_/CX3CR1_low_—monocytes (Mizutani et al. 2012)

**Fig. 1:**
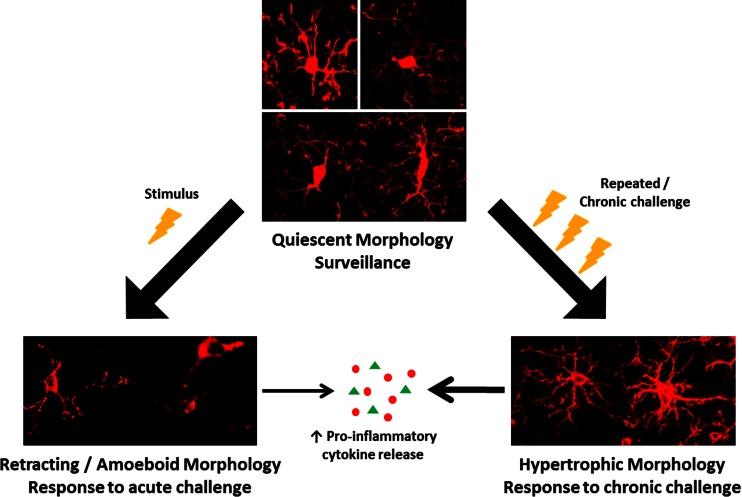
Morphological changes to microglia in response to challenge. In the healthy brain, most microglia display a ‘quiescent’ morphology as they survey the brain. In response to a challenge (*lightning bolt*), such as stress, microglia take on an amoeboid morphology as their processes retract and the soma enlarges. After chronic exposure to CNS challenges, microglia become hypertrophic and primed. Primed microglia produce an exaggerated cytokine response to a subsequent challenge

In the healthy adult central nervous system, microglia have a ramified morphology (Fig. 1), characterised by long, thin processes that enable the microglia to search the local environment for infectious agents or harmful material (Nimmerjahn et al. 2005). When responding to insults, the processes retract, and the cell body enlarges, giving the microglia an amoeboid shape (Cho et al. 2006; Davalos et al. 2005). Chronic inflammatory states are associated with a hypertrophic branch morphology with an enlarged soma (Loane et al. 2014; Hains and Waxman 2006; Stalder et al. 1999). Some of these changes have been shown to occur not only in response to classical inflammatory stimuli, such as infection, but also to psychological stress (Hinwood et al. 2012; Tynan et al. 2010; Sugama et al. 2007; reviewed by Walker et al. 2013).

Here, we aim to review the recent preclinical literature to examine the effects of psychosocial stressors on microglial activity and assess the strength of evidence for a link between stress and microglial changes. For the purposes of this review, we limited our search to studies that utilised the ionised calcium binding adaptor molecule 1 (Iba-1) as a measure of microglial activity, as Iba-1 is expressed by microglia independent of cellular context (Ito et al. 1998), and is widely used, thereby making comparisons across studies possible.

## Methods

### Stress definition

For the purpose of this review, stress was defined as experimental paradigms causing contextual distress to animals. These include physical stimuli such as handling restraint, occlusal disharmony and foot shock, as well as environmental stressors; social isolation, overnight food/water deprivation, social defeat and home cage disruptions (Table 2). These stimuli have been previously validated as effective preclinical models of the social stressors associated with an increased risk of mental illness in humans (Blanchard et al. 2001).

**Table 2:** Preclinical models of psychosocial stress

Stressor	Species	Stress duration	Description	References
Foot shock	C57Bl/6 mice	5 days	Each day, mice were individually placed into a foot-shock box and received 120 shocks (5 s of 0.15 mA/30 s) over the period of an hour. Control animals were placed into the foot-shock box for 1 h/day without any foot shocks.	Brevet et al. (2010)
Chronic restraint	Sprague-Dawley rats, Mongolian gerbils	14–21 days	Animals placed within restrictive wire mesh/Plexiglas environments for up to 6 h/day over the stress duration. Control animals were handled each day and returned to their home cage	Tynan et al. (2010), Park et al. (2011), Yoo et al. (2011), Hinwood et al. (2012, 2013), Kopp et al. (2013)
Occlusal disharmony	ddY mice	1–5 days	Surgical placement of resin on the upper molars increased the vertical dimension of the bite by 0.1 mm. Mice were returned to their home cage for up to 5 days post-surgery with food ad lib. Control mice underwent sham surgery.	Kojo et al. (2010)
Prenatal stress/maternal restraint	Sprague-Dawley rats, C57Bl/6 mice	Gestational day 12/14 delivery	Each day, pregnant females were restrained in restrictive plastic-box environments, with exposure to bright light. Each stress session lasted 45 min and occurred 3 times/day until delivery. Prenatally stressed offspring were assessed when they reached 3–4 months old.	Diz-Chaves et al. (2012, 2013), Ślusarczyk et al. (2015)
Repeated social defeat	C57Bl/6 mice	6 days	Over the 6 days, an intruder (12-month-old retired breeder CD-1 mouse) was placed into the home cage of 3 male C57Bl/6 mice for 2 h/day and asserted dominance over the subordinate resident mice. Control C57Bl/6 mice were housed elsewhere and left without disruption by an intruder.	Wohleb et al. (2011, 2012)
Social isolation	Wistar rats	49 days	Animals were individually housed without physical contact with other rats for the stress duration. Control animals (4–5/cage) were housed in the same room allowing visual, auditory and olfactory stimulation.	Schiavone et al. (2009)
Varying unpredictable stress	Sprague-Dawley rats, C57Bl/6, Kunming mice	2–40 days	Animals were subjected to 2–3 different stressors/day for the duration of the protocol. Stressors include foot shock, restraint, water/food deprivation, forced swim, home cage disruptions, altered light/dark regimes, and overcrowding	Bian et al. (2010), Couch et al. (2013),Giovanoli et al. (2013), Kopp et al. (2013), Kreisel et al. (2014)

### Search approach

**Fig. 2:**
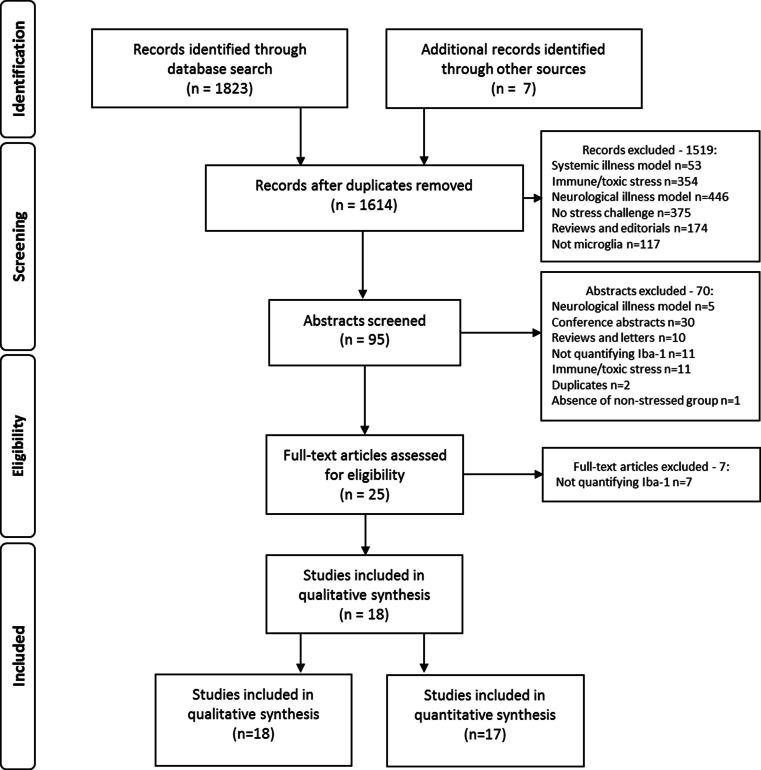
Workflow of study identification and screening process. Systemic-illness models include liver disease, kidney disease and other systemic non-infectious diseases. Neurological-illness models include brain injury or trauma, neurodegenerative disorders and other neurological illnesses

Preclinical studies on the effects of stress on the microglia were identified from electronic searches of PubMed and EMBASE (Fig. 2). We used the following MeSH and free form search terms: stress OR isolation OR defeat OR restraint AND microglia* NOT oxidative NOT spinal NOT retina. The search was performed in June 2015 and limited to studies published between 2000 and 31 May 2015. Reference lists and abstracts of the papers identified by the search were screened to identify additional reports meeting selection criteria. Furthermore, where studies reported incomplete information, authors were contacted to request supplementary data.

### Screen and exclusion criteria

The inclusion criteria were (1) original studies; (2) investigated the effect of psychosocial stress on microglial number, morphology or changes indexed Iba-1; (3) published in English, Spanish, Portuguese or Italian; and (4) published in a peer-reviewed journal.

Studies were excluded if they referred to microglia outside of the brain (i.e. in the spinal cord or retina); if the primary stress was pathological, oxidative or related to neurological (e.g. stroke, nerve damage) or systemic disease, hypo/hyperglycaemia or hypoxia; or if the microglial markers did not include Iba-1 measures. We excluded review articles, letters, comments, multiple publications of the same data and articles that did not publish information necessary to calculate effect sizes or percentage change from baseline.

### Data extraction

We adhered to the ‘Meta-analysis of Observational Studies in Epidemiology’ (MOOSE) reporting guidelines (Stroup et al. 2000). Data were extracted independently by MC and DB, and agreement was confirmed by O.H. For each study, the following variables were recorded: (a) year of publication, (b) type of stress, (c) animal model, (d) brain areas studied, (e) microglia markers and (f) results.

## Results

### Studies

A total of 18 studies were identified that fitted the search criteria. These studies consisted of seven different stress protocols, including social isolation, chronic restraint, repeated social defeat, occlusal disharmony, foot shock, prenatal stress and a combination of varying unpredictable stressors (Table 2). Three species were used in these studies; rats (Wistar and Sprague-Dawley), mice (C57Bl/6, Kunming and ddY) and the Mongolian gerbil. One study (Kreisel et al. 2014) performed varying unpredictable stress under four separate conditions (using two species and two durations of stress exposure). All four conditions were included in the analysis, making a total of 21 stress paradigms examined.

Amongst the 18 studies, three main methodologies were used to measure Iba-1-associated microglial activity: western blot (Ślusarczyk et al. 2015; Schiavone et al. 2009), real-time PCR (Kreisel et al. 2014) and, for the remaining studies, immunohistochemistry techniques. Analysis of Iba-1 staining consisted of region-specific cell counting (Giovanoli et al. 2013; Hinwood et al. 2013; Kopp et al. 2013; Hinwood et al. 2012; Wohleb et al. 2012, 2011; Kojo et al. 2010; Tynan et al. 2010) and/or changes in the percentage area occupied by Iba-1 signal per region (Kreisel et al. 2014; Couch et al. 2013; Diz-Chaves et al. 2013; Hinwood et al. 2013, 2012; Kopp et al. 2013; Bian et al. 2012; Diz-Chaves et al. 2012; Park et al. 2011; Yoo et al. 2011; Brevet et al. 2010; Tynan et al. 2010). The findings are summarised in Table 3. The hippocampus and prefrontal cortex were the most commonly studied regions.

**Table 3:** Effect of stress on microglial activity as measured by Iba-1

Brain region	Stressor	Effect on Iba-1	Species	References
Amygdala	Repeated social defeat	↑↑↑	Mouse	Wohleb et al. (2011, 2012)
	Chronic restraint	→	Rat	Tynan et al. (2010)
Hippocampus	Repeated social defeat, varying unpredictable stress, prenatal stress	↑↑↑	Rat, mouse	Bian et al. (2012), Wohleb et al. (2011, 2012), Ślusarczyk et al. (2015)
	Chronic restraint, prenatal stress	↑↑	Rat, gerbil, mouse	Tynan et al. (2010), Park et al. (2011), Yoo et al. (2011),^a^ Diz-Chaves et al. (2012, 2013)
	Varying unpredictable stress, occlusal disharmony, foot shock	↑	Rat, adult/peripubertal mouse	Brevet et al. (2010), Kojo et al. (2010), Giovanoli et al. (2013), Kreisel et al.(2014)
	Chronic varying unpredictable stress	↓	Rat, mouse	Kreisel et al. (2014)
Nucleus accumbens	Social isolation	↑↑↑	Rat	Schiavone et al. (2009)
	Chronic restraint	↑↑	Rat	Tynan et al. (2010)
Paraventricular nucleus	Repeated Social Defeat	↑↑↑	Mouse	Wohleb et al. (2011, 2012)
	Chronic restraint, chronic varying unpredictable stress	→	Rat	Tynan et al. (2010) and Kopp et al. (2013)
Prefrontal cortex	Repeated social defeat, varying unpredictable stress	↑↑↑	Mouse	Bian et al. (2012), Wohleb et al. (2011, 2012)
	Prenatal stress	↑↑	Rat	Ślusarczyk et al. (2015)
	Social isolation, chronic restraint, varying unpredictable stress	↑	Rat, mouse	Schiavone et al. (2009), Tynan et al. (2010), Hinwood et al. (2012, 2013), Couch et al. (2013), Kopp et al. (2013)
	Varying unpredictable stress	→	rat, mouse	Giovanoli et al. (2013), Kopp et al. (2013), Kreisel et al. (2014)
Ventral tegmental area	Chronic restraint	→	Rat	Tynan et al. (2010)

Measure of change in Iba-1 activity by stress protocol relative to unstressed control animals: no significant change (→), >5 % decrease (↓), 5–30 % increase (↑), 30–70 % increase (↑↑) and >70 % increase (↑↑↑)

^a^As reported by authors

### Effect of psychosocial stress on Iba-1 activity within the hippocampus

The effect of stress on Iba-1 activity within the hippocampus was investigated by 11 of the studies, incorporating 13 different stress protocols. These included foot shock (one study), occlusal disharmony (one study), chronic restraint (two studies), repeated social defeat (two studies), prenatal stress (three studies) and varying unpredictable stress (four protocols in two studies). The variable unpredictable stress regimes comprised a number of random home cage disruptions, including tilting, background noise, mistimed light exposure, restraint and foot shock over 2–4 days (two protocols) or 10–35 days (two protocols).

When compared with unstressed control animals, 11 of the 13 protocols in all 11 studies reported significantly increased Iba-1 activity within the hippocampus. The magnitude of these increases varied across protocols, with repeated social defeat resulting in a more than 2-fold increase in the number of Iba-1-positive cells present (Wohleb et al. 2011), whereas occlusal disharmony, foot shock and varying unpredictable stress led to more moderate increases of 16–27 % (Table 3). The changes in Iba-1 were not consistent across the whole hippocampus. Kreisel et al. (2014) demonstrated a 30 % increase in Iba-1 messenger RNA (mRNA) and ∼50 % increase in Iba-1 staining in the dentate gyrus of rats and mice, respectively, following 2–4 days of variable unpredictable stress. In contrast, after 5 days of occlusal disharmony, Kojo et al. (2010) did not observe any significant changes in the number of Iba-1-positive cells within the dentate gyrus of mice, but saw increased Iba-1-positive cell counts in the CA1 region of the hippocampus. This was in line with the 2-fold increase in Iba-1 staining reported in the CA1 region following 40 days of varying unpredictable stress by Bian et al. (2012).

Furthermore, studies of prenatal stress by Diz-Chaves et al. (2012, 2013) found sex differences within the dentate gyrus; prenatally stressed female offspring showed increased number of Iba-1-positive cells in the dentate gyrus whereas males did not show a significant change. Despite no change in the number of cells, the Iba-1 signal was significantly increased within the dentate gyrus of prenatally stressed male mice. No significant changes in Iba-1 activity were reported in the CA1 region of male mice.

Interestingly, Kreisel et al. (2014) found differences in Iba-1 signal between their two protocols. Whereas short-term exposure (2–4 days) to varying unpredictable stress led to increased hippocampal Iba-1 signal, longer exposure (35 days) was observed to decrease Iba-1 mRNA in the dentate gyrus of both rats (35 % decrease) and mice (27 % decrease).

Only one study examined the effect of stress within the CA3 region of the hippocampus. Tynan et al. (2010) found that 14 days of chronic restraint in Sprague-Dawley rats resulted in a moderate increase (∼29 %) in the number of Iba-1-positive cells. There was however, an increase of approximately 60 % in the density of Iba-1 signal within the CA3 region of the same subjects.

### Effect of psychosocial stress on Iba-1 activity within the prefrontal cortex

Changes in Iba-1 signal within the prefrontal cortex were examined by 13 of the studies using five different stressors. These included social isolation (one study), prenatal stress (one study), repeated social defeat (two studies), chronic restraint (four studies) and varying unpredictable stress (five studies).

Social isolation of adult rats for 35 days resulted in ∼15 % increase in Iba-1-positive protein in the prefrontal cortex (Schiavone et al. 2009). This was comparable with the increase in Iba-1-positive cell counts reported by Tynan et al. (2010), following chronic restraint of rats for an hour a day for 14 days. Notably, these rats were also individually housed at the start of the study (and acclimatised for at least 7 days). Further analysis by Hinwood et al. (2013) found that chronic restraint led to distinct morphological changes, with increased ramification of the microglia. A more recent study by Kopp et al. (2013), which restrained rats for only 30 min a day for 14 days, found that while there was a significant increase in the percentage area of Iba-1 signal throughout the prefrontal cortex (11 % increase), there was no significant change in the number of Iba-1-positive cells. Using an alternative stressor, the varying unpredictable stress protocol, Kopp et al. (2013) found no significant changes in Iba-1 activity in the prefrontal cortex of rats. In mice, the varying stress protocol has produced similarly varied responses in prefrontal cortex Iba-1 activity (Kreisel et al. 2014; Couch et al. 2013; Bian et al. 2012). The greatest changes within the prefrontal cortex were produced in mice undergoing repeated social defeat for 6 days, which resulted in a 2-fold increase in Iba-1 staining (Wohleb et al. 2012, 2011).

### Psychosocial stress affects Iba-1 activity in other brain areas

In addition to the hippocampus and prefrontal cortex, five studies examined the effect of psychosocial stress on Iba-1 activity in other areas of the brain.

Within the nucleus accumbens, both chronic restraint and social isolation of rats resulted in significant increases in Iba-1 activity by ∼20–40 % (Iba-1 cell counts and density) and 73 % (protein density), respectively (Tynan et al. 2010; Schiavone et al. 2009).

Two studies by the same lab used repeated social defeat in mice for 6 days and found significant increases in the number of Iba-1-positive cells in the amygdala (1.6–2.4-fold) and paraventricular nucleus (1.5–2.8-fold) of the hypothalamus (Wohleb et al. 2012, 2011). These findings were in contrast to those produced by chronic restraint and varying unpredictable stress models which found no significant changes in Iba-1 within the amygdala (Tynan et al. 2010) or paraventricular nucleus (Kopp et al. 2013; Tynan et al. 2010).

Lastly, only the study by Tynan et al. (2010) examined the ventral tegmental area and found that chronic restraint in rats did not lead to any significant changes in Iba-1 when compared with control animals. This included both in the number of Iba-1-positive cells as well as the percentage change in area covered by Iba-1 signal.

## Discussion

We identified 18 studies showing that psychosocial stress increases expression of the microglial marker Iba-1 in at least one of the brain regions examined by each study and in many cases, in multiple brain areas. These findings support the hypothesis that exposure to psychosocial stress increases the risk of a number of mental illnesses through the activation of microglia and other centrally mediated immune responses (Frick et al. 2013; Nair and Bonneau 2006).

### Potential mechanisms

The expression of glucocorticoid receptors in the brain is greatest within the hippocampus, making it an area particularly sensitive to stress-inducing stimuli (McEwen et al. 1992; Aronsson et al. 1988). Similarly, the prefrontal cortex has a high number of glucocorticoid binding sites and has been implicated in the body’s response to stress-signalling via mediation of the hypothalamic-pituitary adrenal (HPA) axis (Gold 2015; Diorio et al. 1993). Furthermore, several studies have demonstrated the negative impact of similar stress protocols on learning and memory (Lemaire et al. 2000; Lordi et al. 1997). Hence, it is not surprising that of the 18 studies identified, 13 investigated stress-associated changes in microglial activity within the hippocampus and 12 within the prefrontal cortex.

In rodents, activation of the HPA axis following stress results in a surge of circulating corticosterone (Meaney et al. 1994). In view of the high number of glucocorticoid receptors present in the hippocampus and prefrontal cortex, these regions are likely to be particularly sensitive to the corticosterone surge, leading to indirect effects on microglia in these regions. In addition, as microglia express both glucocorticoid and mineralocorticoid receptors (Sierra et al. 2008), there may be direct effects of stress-induced corticosterone surges on microglia. Increased Iba-1 signal within the hippocampus was reported by all 13 studies, although one of these studies also reported decreased Iba-1 when utilising a longer stress duration paradigm (Kreisel et al. 2014). These differences in Iba-1 may reflect changes in HPA-axis regulation, whereby in response to chronic stress, negative feedback mechanisms via glucocorticoid receptor activation leads to reduced corticosterone release (Adzic et al. 2009; Sánchez et al. 1998).

The response of the immune system is known to change with both duration and frequency of stress exposure as the organism attempts to adapt (McEwen 2000). One mechanism by which this occurs is through priming of the nucleotide-binding domain, leucine-rich repeat, pyrin domain-containing proteins-3 (NLRP3) inflammasome, a component of myeloid cells including macrophage and microglia involved in cleaving pro-IL-1β into its active form (Martinon et al. 2002). A recent study by Frank et al. (2014) demonstrated that chronic treatment with glucocorticoids dose-dependently increased expression of NLRP3 and Iba-1 within the hippocampus. Furthermore, subsequent immune challenge with LPS resulted in a potentiated microglial response indicative of priming.

In the study by Giovanoli et al., which found no change in Iba-1 within the prefrontal cortex but moderate increases in the hippocampus, a suggested explanation may be through permanent modifications in the HPA-axis caused by maternal stress (Baquedano et al. 2011; Brunson et al. 2001a, b). Maternal stress acts, at least partly, via changing glucocorticoid action in the placenta impacting the behaviour of offspring (Räikkönen et al. 2015). Prenatal stress also increases IL-1β mRNA levels in the hippocampus and the proportion of microglia cells with large somas during adult life of rodents (Diz-Chaves et al. 2012). Of particular interest is the subsequent immune response of the prenatally stressed offspring. A second systemic immune stimulus given later in life, such as exposure to lipopolysaccharide (LPS), exacerbates the immune response and corticosterone levels in the adult rats (Diz-Chaves et al. 2013). The importance of this finding and the impact of early-life stress exposure on subsequent later-life behaviours and health have only recently been explored (Réus et al. 2015).

In addition to the release of glucocorticoids, psychosocial stress may exert direct effects on microglial activity through other pathways, including the sympathetic release of noradrenaline and subsequent activation of β-adrenergic receptors. This activation during stress has been associated with increased pro-inflammatory cytokine (IL-1β) expression within the central nervous system, leading to potential priming of microglia (Johnson et al. 2008, 2005, 2013; McNamee et al. 2010). Furthermore, using the β-adrenergic antagonist propranolol, Wohleb et al. (2011) effectively blocked the increased Iba-1 signal induced by social defeat in multiple brain regions including the hippocampus and prefrontal cortex.

### Implications for understanding the pathoaetiology of mental illnesses

The general trend resulting from nearly all of the stress paradigms examined suggest that exposure to stress can lead to significant increases in microglial activity. This finding could have implications for mental health research, with studies indicating increased microglial activity prevalent amongst patients with depression, anxiety and schizophrenia (Bloomfield et al. 2015; Frick et al. 2013). Therefore stress, as a potential stimulus of microglial activity, may contribute to the development of various mental disorders.

A number of studies show an association between greater microglial activity and behavioural outcomes that are proxies for depressive and anxiety-like symptoms in animal models. Studies by Wohleb (2011, 2012) using the social defeat model showed anxiety-like behaviour positively associated with microglial activity. Couch et al. (2013), using a model that combined social defeat, restraint and tail suspension, observed a pro-inflammatory profile and heightened microglial activity associated with the development of stress-induced anhedonia in mice susceptible to depression following exposure to stress. These changes in the microglial profile were not present in ‘resilient’ animals that did not develop depressive symptoms following the stress protocol.

Social defeat has been used as a behavioural correlate for depression, as it provides a comprehensive representation of depressive-like symptoms that can be measured in animals (Hollis and Kabbaj 2014). Similarly, social isolation may be used as a stress paradigm in weaning, adolescent or adult rats, and has been associated with reduced preference for sucrose (Krishnan and Nestler 2011) and reduced swimming during the forced swim test (Djordjevic et al. 2012), both depressive-like symptom paradigms. Though the nature of stress and adversity factors linked to mental illness is complex (Bagot et al. 2014; Howes and Murray 2014), the animal models used in the studies reviewed provide a useful indicator that a range of psychosocial stressors, including both individual (e.g. restraint) and social (e.g. social isolation and defeat), are capable of increasing microglial activity. Moreover, anti-inflammatory medication has been found to reduce depression-like behavioural deficits and reduce microglial activity (Burke et al. 2014; Hinwood et al. 2012; Henry et al. 2008), providing further support for a mechanistic link between microglial activity and psychiatric symptoms.

**Fig. 3:**
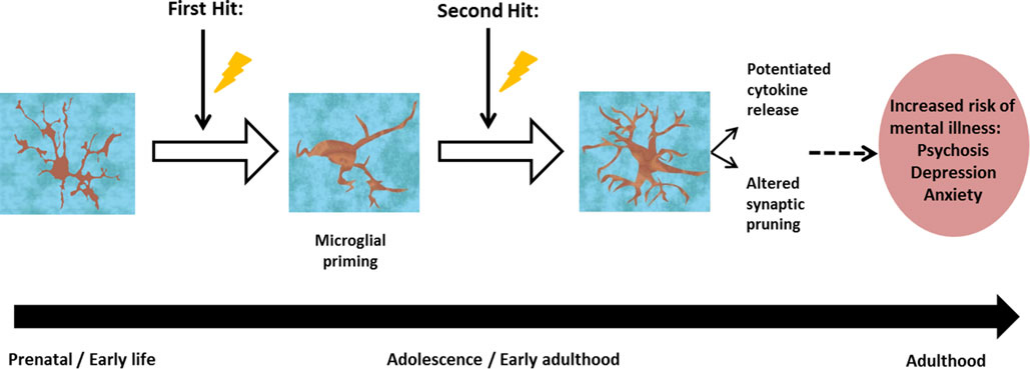
The ‘two-hit’ hypothesis: exposure to prenatal/early-life stress (*lightning bolt*) may act to prime microglia within the CNS so that a subsequent challenge later in life, either in adolescence or adulthood invokes a potentiated microglial response, leading to an increased risk of developing a mental illness

Three of the studies included in our search specifically examined the effect of prenatal stress on microglial changes and found significant increases in Iba-1 activity (Ślusarczyk et al. 2015; Diz-Chaves et al. 2013, 2012). Of these, the two by Diz-Chaves et al. (2012, 2013) found potentiated responses in prenatally stressed animals that were subsequently given an immune challenge in adulthood. Similarly, another study found that a prenatal immune challenge left mice more susceptible to markedly potentiated responses to stress during their peripubertal period (Giovanoli et al. 2013). The effects of maternal/early-life stress on microglial activity closely resembled that caused by pathogenic immune challenge in rodents (Juckel et al. 2011; Bland et al. 2010). Such studies support the idea of stress leading to microglial priming, where an initial stimulation early in life primes microglia, leading to an exaggerated response of the microglia to a second inflammatory stimulus following initial stimulation (Frank et al. 2007). This underlies the ‘two-hit’ hypothesis (Fig. 3). This hypothesis proposes that early-life stress sensitises microglial cells so that there is an exaggerated elevation of microglial activity to stress in late adolescence or adulthood, leading to brain changes that underlie the development of a mental disorder (Hickie et al. 2009). As demonstrated by the preclinical studies identified here, many forms of psychosocial stress are able to stimulate microglial activity throughout the brain, and in several cases, prenatal stress exposure was sufficient to cause lasting changes in microglial response.

It is beyond the scope of this review to examine the effects of immune stressors such as LPS and cytokine administration on microglial activity, which have been described extensively elsewhere (Harry 2013; Juckel et al. 2011; Dantzer et al. 2008) and clearly show up regulation of microglial markers. A number of the studies included in our search demonstrated that while stress alone is sufficient to increase Iba-1-activity in several areas of the brain, a later immune challenge (‘second-hit’) potentiated the Iba-1 signal (Ślusarczyk et al. 2015; Diz-Chaves et al. 2012, 2013; Giovanoli et al. 2013; Wohleb et al. 2012; Yoo et al. 2011).

### Limitations of current evidence

There was notable variability in responses seen across the studies. This may simply be the result of methodological differences; each of the stress regimes differs in terms of their severity and duration. There is also some overlap of stress protocols; prior to the chronic restraint protocol used by Tynan et al. (2010), animals were individually housed for a minimum of 1 week to acclimatise. It is difficult then to separate the effects of chronic restraint from the effects of social isolation, which were described by Schiavone et al. (2009). However, it is interesting to note, that within the same lab, undergoing an identical stress regime; we observed that Wohleb et al. (2011, 2012) reported variability of approximately 1.5-fold difference in the magnitude of the control vs. stressed groups (both drug naïve) between the two studies. This variability may result from a number of factors, including the small group sizes of both studies (*n* = 3–5), slight differences in the methodologies; mice were handled and given vehicle administrations either just prior to social defeat or 14 h after social defeat cycles or possibly as a result of the stressor itself. Here, repeated social defeat introduced an intruder (typically a retired breeder) into an established colony of mice, leading to the establishment of dominance over the original colony (i.e. aggression). The physically damaging nature of this stressor can lead to an injury triggering the immune system, together with the psychosocial stress response, which may account for increased variability across studies.

The study by Kreisel et al. (2014) demonstrates that stress duration is an important consideration. Exposure to variable stressors for an acute period (2 days) resulted in upregulated Iba-1 expression via microglial proliferation. In contrast, chronic exposure to this same regime led subsequently to significant decreases within the hippocampus as a result of increased microglial apoptosis. In addition to this, variations in response and magnitude may be due to species and sex differences, the latter being demonstrated by the two studies by Diz-Chaves et al. (2012, 2013), which showed slight differences in Iba-1 expression within specific areas of the mouse hippocampus. A recent study by Bollinger et al. (2015) also showed sex differences in Iba-1 expression in the rat prefrontal cortex. While there was no difference in the total number of microglia, morphological studies showed unstressed female rats had a greater proportion of primed to ramified microglia than males, suggesting greater basal microglial activity. In response to restraint stress, the proportion of primed microglia decreased in female rats, indicating an inhibitory effect of stress, whereas there was no significant change observed in the males.

Several markers for microglia have been identified (Table 1). Of these, Iba-1 is one of the most commonly used given its CNS selectivity for microglia and infiltrating macrophage (Wohleb et al. 2013). Hence, Iba-1 has been predominantly used to determine information regarding the morphological changes and distribution of microglia in response to pathological and non-pathological challenge. In healthy environments, the majority of microglia have a ramified process morphology with multiple branches that extend as sensors for potential challenges (Nimmerjahn et al. 2005). When a challenge is present, these cells become amoeboid-like, with enlarged soma, fewer branches and processes (Davalos et al. 2005; Stence et al. 2001). However, these stimulated microglia are a heterogeneous mix of cells, which functionally can be divided into pro-inflammatory (M1) and anti-inflammatory (M2) states and, more recently, other phenotypes have been identified (Crain et al. 2013). Of these Mhem, Mox, and M4 show major immunomodulatory and anti-inflammatory properties (Hu et al. 2015; Naito et al. 2014; Moore et al. 2013). In responding to a challenge, M1 microglial cells are the initial source of pro-inflammatory cytokines, such as IL-1β and IL-6, whereas M2 microglia respond secondary, leading to the release of anti-inflammatory signals to restore the system back to normal. Alone therefore, Iba-1 measures provide morphological but limited functional information regarding the CNS response to stress challenges. Alongside Iba-1, several of the studies included additional measures such as cytokine levels to ascertain the inflammatory effects (pro- vs. anti-) of stress. These findings were in line with previous studies of acute stress on pro-inflammatory cytokine induction (Himmerich et al. 2013; O’Connor et al. 2003).

### Future directions and implications for new treatments

Given the growing body of preclinical evidence indicating that stress leads to microglial priming, we suggest that further work in this area focus on two areas. First, preclinical studies are needed to elucidate mechanisms underlying priming and ways to block subsequent effects of stress. Second, clinical studies are needed to determine if priming occurs in humans.

Notwithstanding this, the evidence that stress both increases the risk of a number of mental disorders and leads to elevated microglial activity suggests that targeting this mechanism could treat or even prevent some mental disorders. Some of the studies analysed here have found that treatment with minocycline reversed some stress-induced microglial changes (Hinwood et al. 2012, 2013). Recent clinical studies using minocycline as an adjunctive treatment for patients with schizophrenia have shown promising early results, particularly in the treatment of negative and cognitive symptoms (Chaudhry et al. 2012; Levkovitz et al. 2010). Other promising targets include inhibitors of the enzyme cyclooxygenase-2 (COX-2), which is involved in inflammation signalling (Keller et al. 2013; Müller et al. 2013). Celecoxib, a COX-2 inhibitor given alongside amisulpride improved the therapeutic outcome of early-diagnosed schizophrenic patients when compared with amisulpride alone in a recent 6-week double-blind study (Müller et al. 2010). The findings we review here suggest that targeting inflammation-pathways of susceptible individuals may help to increase resilience to future stress and attenuate the exaggerated responses of primed microglia.

The effects of psychosocial stress on Iba-1 activity were predominantly examined within the prefrontal cortex and hippocampus. However, Tynan et al. (2010) comprehensively examined multiple brain regions from rats exposed to chronic restraint and found significant changes throughout the CNS. Nevertheless, it remains unclear to what extent the changes in Iba-1 activity occur globally, or are regionally selective following the other forms of psychosocial stress used. Future studies to explore this question and the pathways by which different brain regions receive/respond to stress signals will prove useful.

Another important area of focus for future research is to test the link between microglial priming, two-hit susceptibility and later development of mental health disorders. Microglial priming has been studied in relation to neurodegenerative disorders (Perry and Holmes 2014), and a recent morphometry study has found evidence of increased microglial priming in middle-aged patients with depression who died by suicide compared with healthy controls (Torres-Platas et al. 2014). Further developments in the field of microglial activity and priming have the potential for making important changes in our understanding and treatment of mental disorders in the future.

## Conclusions

Our systematic review indicates that stress exposure reliably leads to a microglial response in hippocampus and prefrontal cortical regions, and there is some evidence to suggest similar effects in other regions as well.
